# Analysis of gut microbiota in three species belonging to different genera (*Hemitragus*, *Pseudois*, and *Ovis*) from the subfamily Caprinae in the absence of environmental variance

**DOI:** 10.1002/ece3.7976

**Published:** 2021-07-31

**Authors:** Guolei Sun, Tian Xia, Qinguo Wei, Yuehuan Dong, Chao Zhao, Xiufeng Yang, Lei Zhang, Xibao Wang, Weilai Sha, Honghai Zhang

**Affiliations:** ^1^ College of Life Science Qufu Normal University Qufu China

**Keywords:** 16S rRNA gene, Caprinae, gut microbiota, high‐throughput sequencing, host–microbiome interaction

## Abstract

This study aimed to identify the effects of host species on the gut microbial flora in three species (*Hemitragus jemlahicus*, *Pseudois nayaur*, and *Ovis orientalis*) from the subfamily Caprinae, by excluding the impact of environment factors. We investigated the differences in intestinal flora of three species belonging to Caprinae, which were raised in identical conditions. Fecal samples were collected from tahr, mouflon, and bharal, and the V3–V4 region of the 16S ribosomal RNA gene was analyzed by high‐throughput sequencing. The analysis of 16S rRNA gene sequences reveals that fecal samples were mainly composed of four phyla: Firmicutes, Bacteroidetes, Spirochaetes, and Proteobacteria. The most abundant phyla included Firmicutes and Bacteroidetes accounting for >90% of the bacteria, and a higher Firmicutes/Bacteroidetes ratio was observed in tahrs. Moreover, significant differences existed at multiple levels of classifications in the relative abundance of intestinal flora, differing greatly between species. Phylogenetic analyses based on 16S rRNA gene indicated that mouflon is closely related to bharal, and it is inconsistent with previous reports in the species evolutionary relationships. In this study, we demonstrated that the gut microbiota in tahr had a stronger ability to absorb and store energy from the diet compared with mouflon and bharal, and the characteristics of host–microbiome interactions were not significant.

## Graphical abstract

We investigated the differences in intestinal flora of three species belonging to Caprinae living in the same environment and raised in identical conditions. We demonstrated that the microbiota in tahr had a stronger ability to absorb and store energy from the diet compared with mouflon and bharal, and the characteristics of host–microbiome interactions were not significant.

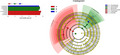

## 1 INTRODUCTION

Microbes are widespread in nature and exist in all potential habitats (Alnahhas et al., 2020). A vast number of microorganisms inhabited in mammalian intestines constitute a complex microecological system (Heintzbuschart & Wilmes, 2017; Thursby & Juge, 2017). The gut microbiota can be shaped by various factors, such as diet, host genetics, and medication (Wu & Gao, 2015). Researches suggest that the composition and function of intestinal microbiota can be strongly influenced by diet and medication, and diet has been demonstrated to play a deterministic role in shaping the gut microbiota (Crommen et al., 2020; Jackson et al., 2018; Rothschild et al., 2018; Turpin et al., 2016). In the same way, host physiological processes, such as host metabolism, immune responses, and energy balance, can also be influenced and regulated by host–gut microbiota interactions (Huttenhower et al., 2012; Macke et al., 2017; Turnbaugh et al., 2006). In addition, the imbalance of gut microbiota causes intestinal inflammation and metabolic properties (Tremaroli & Backhed, 2012; Yang et al., 2020). For example, obesity and type 2 diabetes are demonstrated to be associated with gut microbial imbalance (Gérard, 2016; Sircana et al., 2018). Understanding the effects of factors on microbial communities (microbiomes) is highly important, which can reduce the risk of potential diseases related to the gut microbiota.

Previous researches have shown that the composition of the gut microbiota varies among different races around the world (de la Cuesta‐Zuluaga et al., 2018; Deschasaux et al., 2018). Researches focusing on gut microbiota with Chinese indicated that the composition of the microbial communities was significantly different across ethnic populations in the same geography, with a higher bacterial diversity and relative abundance of Prevotella in Tibetans (Li et al., 2016; Liu et al., 2020). However, researches on drosophila and mosquitoes showed that the effects of host species make no or much smaller differences in gut microbiota, as compared with developmental stage or geographical location (Bascuñán et al., 2018; Martinson et al., 2017). Furthermore, evidence suggested that host phylogeny is related to gut microbial differences (Groussin et al., 2017; Li et al., 2017; Ochman et al., 2010). Another study on chimpanzees and gorillas suggested that sympatric species had a more similar microbial community structure, and the different populations of the same species in different geographical locations showed a larger difference (Moeller et al., 2013). In addition, a study on seven Cervinae species under the same feeding conditions indicated that microbiomes divergence and host phylogeny had a certain correlation, but this correlation was not always consistent (Li et al., 2018).

The herbivores distinct from omnivore and carnivore, with the highest bacterial diversity of gut microbiota (Ley et al., 2008; Price et al., 2012). Over half of energy intake of ruminants arise from cellulose degradation, while this percentage is as low as 10% in humans (Bergman, 1990). This study focused on three species belonging to Caprinae, and effects of dietary differences and environmental variations were eliminated under the same feeding condition. Moreover, these all three species readily adapt to the high altitude with cold and harsh environments and have abilities in cellulose degradation (Hoefs, 1985; King & Forsyth, 2021; Schaller, 1998). In addition, host phylogeny and divergence time could even be predicted by gut microbiota, and it brings new insights into the potential mechanisms and interrelations between host and microorganisms (Li et al., 2017). Although interrelations between host species and gut microbiota are important, few studies on rare and protected ruminants (Li et al., 2019; Naya & Karasov, 2011; Przybylo et al., 2019; Zhao et al., 2019). Our findings provide new insights into changes in the microbial flora and host phylogeny and provide strategies for protection.

## 2 MATERIALS AND METHODS

All animal experiments in this study were performed in accordance with the recommendations of the Guide to Animal Experiments of the Ministry of Science and Technology (Beijing, China). Ethics approval for this research was obtained from the Qufu Normal University Institutional Animal Care and Use Committee (Permit Number: QFNU2018‐031).

### 2.1 Sample collection and phylogeny of host species

We collected a total of 20 fresh fecal samples from Ji'nan Wild Zoo for sampling, with tahr (group A, *n* = 11), mouflon (group B, *n* = 5), and bharal (group C, *n* = 4). All samples were from healthy and adult individual (age 3–8 years) and were not treated with any antibiotics for 3 months or less prior to collection. The animals in this study lived in the same environment with the same feeding conditions, and the major food categories included cotton‐grass, alfalfa, and pellet feed. All samples were collected immediately at the time of early morning after excretion. Samples were frozen in liquid nitrogen rapidly and then transferred to the laboratory and stored at −80°C until needed. Simulated phylogeny of five species were obtained using TimeTree database (http://www.timetree.org) (Hedges et al., 2015).

### 2.2 DNA extraction and 16S rRNA amplicon sequencing

DNA was extracted from fecal samples with the QIAamp^®^ Stool Mini Kit (Qiagen) using the manufacturer's protocols. DNA concentrations were measured using a *NanoDrop* 2000 *UV‐Vis* spectrophotometer (Thermo Fisher Scientific).

The V3–V4 region of the microbial 16S rRNA gene was amplified using gene‐specific primers, (Forward primer 5′ CCTAYGGGRBGCASCAG; reverse primer 5′ GGACTACNNGGGTATCTAAT). The polymerase chain reaction (PCR) reaction was carried out in a 50 µl mixture volume containing 5 µl template DNA, 5 µl of each primer, 25 µl KAPA HiFi HotStart Ready Mix (KAPA Biosystems), and 10 µl ddH_2_O. The PCR conditions were used: 95°C for 10 min, followed by 25 cycles of 94°C for 30 s, 55°C for 30s, and 72°C for 30 s, and a final elongation step at 72°C for 5 min. PCR products (∼410 bp) were resolved on 1% agarose gels and then purified with AMPure XP beads following the PCR purification kit. A sequence library of amplicons was constructed using TruSeq DNA PCR‐free Sample Preparation Kit (Illumina). Paired‐end sequencing was performed using an Illumina MiSeq platform (Illumina). The sequence data are available in the NCBI Sequence Read Archive under accession number PRJNA664214, PRJNA511517.

### 2.3 Sequence processing and bioinformatic analyses

Raw reads from the Hiseq sequencing were processed and assembled to control the quality of raw data with the following criteria. Sequencing data were first trimmed using FLASH (version 1.2.7) and QIIME (version 1.7.0) to cut off the barcodes and primer sequences and then transformed into raw tags (Caporaso et al., 2010; Magoč & Salzberg, 2011). Chimera were identified and removed using UCHIME algorithm, and effective tags were obtained (Edgar et al., 2011). Quality sequences were clustered into operational taxonomic units (OTUs) at a similarity cutoff value of 97% following the UPARSE (version 7.0.1001) pipeline (Edgar, 2013). RDP classifier algorithm (version 2.2) was applied to assign taxonomy to a species level with the GreenGene Database (McDonald et al., 2012; Wang et al., 2007).

Alpha diversity was measured by five indices (ACE, Chao1, Goods coverage, Shannon, Simpson, and Observed species) with QIIME (version 1.7.0). Venn diagram was constructed to define unique and shared OTUs between groups. Rarefaction and rank–abundance curves were used to indicate the bacterial diversity and species richness of the samples. Tukey's test and Wilcoxon rank‐sum test were performed for statistical analysis. The clustering of different samples was demonstrated by principal component analysis (PCA), principal coordinate analysis (PCoA), and nonmetric multidimensional scaling (NMDS) diagrams with R version 2.13.1. The dendrograms of similarity matrices with weighted Unifrac were performed by unweighted pair group method with arithmetic mean (UPGMA). Linear discriminant analysis (LDA) effect size (LEfSe) analysis was used to find biomarkers at all taxonomic levels in different groups (Segata et al., 2011).

### 2.4 Statistical analysis

The differences between groups were assessed using Student's *t* test with a significance level set at *p* < 0.05. Analysis of similarity (ANOSIM) was employed to evaluate the statistically significant differences in intestinal microbiota between groups with the R vegan package (Anderson, 2001). To identify differences of microbial diversity between groups, MRPP was used to test the composition and structure of microbial communities in grouped samples by R version 2.13.1.

## 3 RESULTS

### 3.1 Phylogeny of host species and DNA sequencing

**FIGURE 1:**
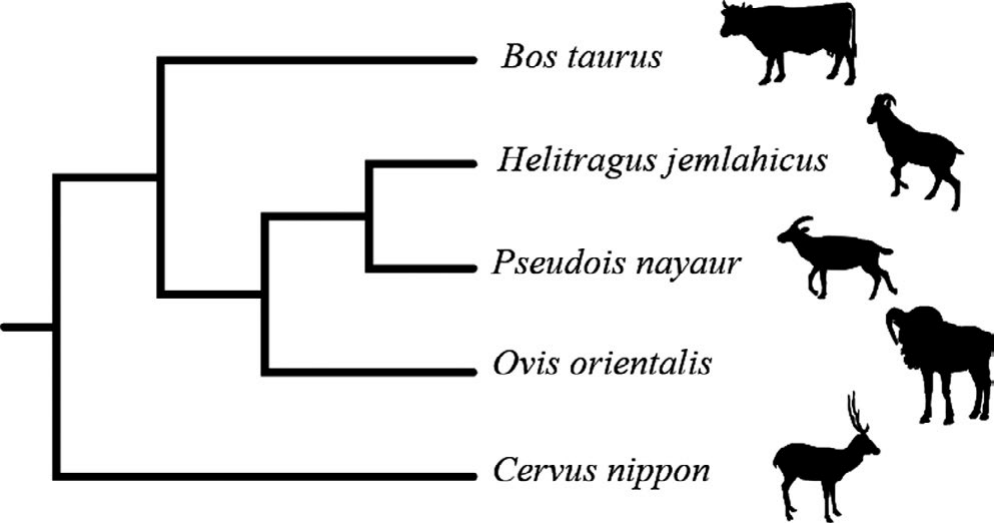
Phylogenic relationships between species (cow, tahr, bharal, mouflon, and sika deer). Phylogenetic tree showing that tahr and bharal branches were first grouped together and then clustered with mouflon

The cladogram was obtained using the TimeTree database, and the phylogenetic tree showed that the tahr was firstly grouped with bharal, and then clustered with mouflon. (Figure 1). A total of 1,543,333 raw reads was obtained from the 20 samples, and the V3–V4 region of the 16S rRNA gene was 412 bp with average length. Raw reads were filtered by QIIME, and a total of 1,365,306 clean reads were obtained, and the Q30 values of the clean data were required to be greater than 96.88%. An average of 68,265 (range, 45,780–75,670) reads mapped per sample and the median was 70,591.

### 3.2 Bacteria composition and relative abundance

**FIGURE 2:**
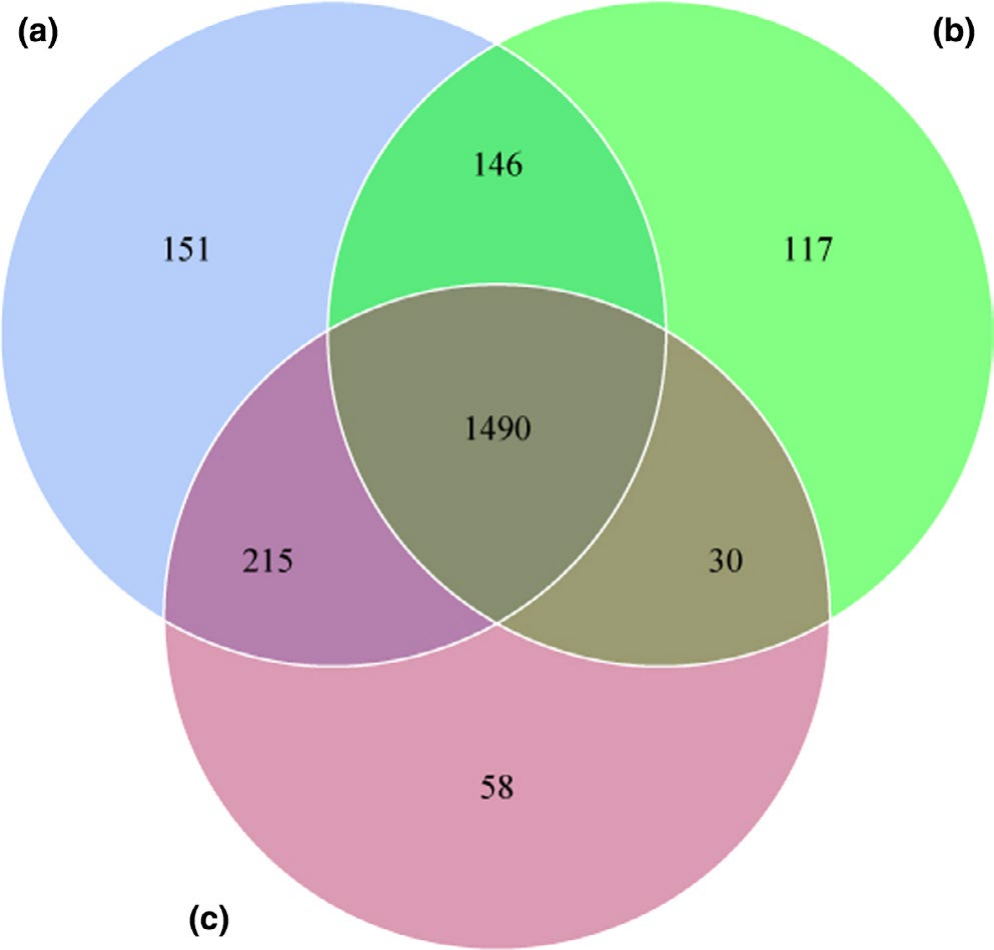
Venn diagram showing the specific and shared OTUs of different species

Raw reads were filtered by QIIME, and a total of 27,828 operational taxonomic units (OTUs) were obtained from 20 samples and then clustered into 2,223 unique OTUs based on 97% sequence similarity. The average number of OTUs per species was inconsistent with 2,002 in tahr, 1,783 in mouflon, and 1,793 in bharal, and 1,490 OTUs were shared between these three species (Figure 2).

Statistical analysis was conducted based on taxonomical data and 22 phyla, 45 classes, 83 orders, 135 families, 273 genera, and 87 species were detected in all samples. At the phylum level, relative abundances of the top five phyla detected in tahr (group A) included Firmicutes (66.11%), Bacteroidetes (25.91%), Spirochaetes (2.23%), Proteobacteria (2.18%), and Cyanobacteria (0.89%). Top five phyla in mouflon (group B) included Firmicutes (57.17%), Bacteroidetes (33.68%), Proteobacteria (2.15%), Spirochaetes (1.64%), and Cyanobacteria (1.45%). Top five phyla in bharal (group C) included Firmicutes (57.22%), Bacteroidetes (33.48%), Spirochaetes (2.40%), Proteobacteria (2.31%), and Fibrobacteres (1.31%). It is noteworthy that the relative abundance of the four phyla account for over 94% of the bacterial community. Statistical analysis showed significant differences between groups. The relative abundance of Firmicutes was significantly higher while Bacteroidetes was significantly lower in tahr than the other two species (*p* < 0.05). Our results also indicated highly significant differences in the relative abundance of Spirochaetes between mouflon and bharal (*p* < 0.01).

**FIGURE 3:**
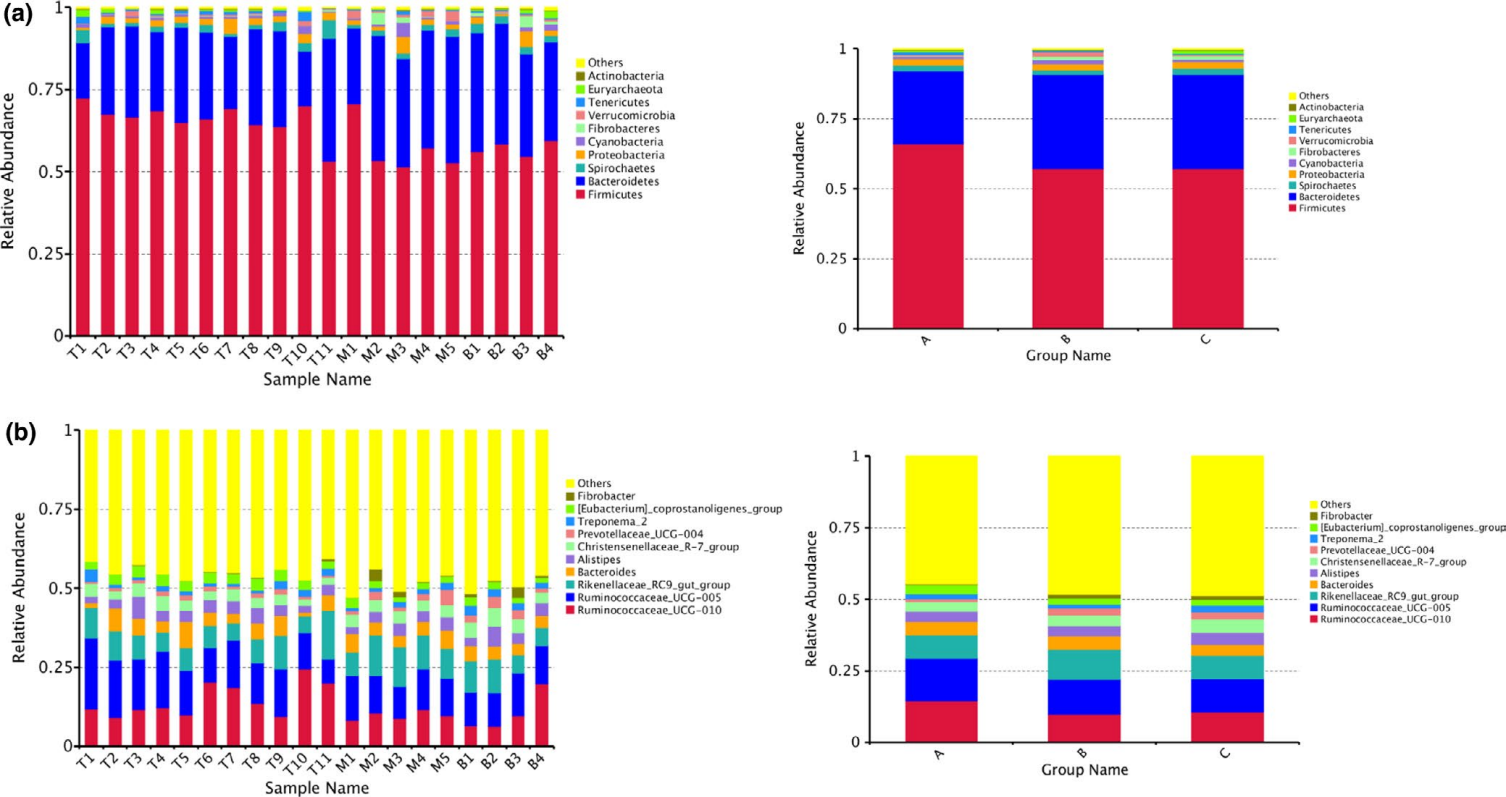
Relative abundance of the top 10 bacterial phyla (a) and genera (b) for three species, and both samples and groups are shown, respectively

We then explored the differences in bacterial communities at the genus level between groups, and the dominant bacterial genera in fecal samples included Ruminococcaceae_UCG‐010, Ruminococcaceae_UCG‐005, Rikenellaceae_RC9_gut_group, Bacteroides, Alistipes, Christensenellaceae_R‐7_group, Prevotellaceae_UCG‐004, Treponema_2. Genera Ruminococcaceae_UCG‐010, Akkermansia, and Phocaeicola were significant difference between tahr and mouflon, while genera Christensenellaceae_R‐7_group and Prevotellaceae_UCG‐004 were significant difference between tahr and bharal. Also, there was a significant difference in genera Treponema_2, Prevotella_1, and Akkermansia between mouflon and bharal. The relative abundance of genus Akkermansia was significantly higher for mouflon group than others. Figure 3a shows the relative abundances of the top 10 phyla, and Figure 3b shows the relative abundances of the top 10 genera. In addition, relative abundance of the top 10 classes, orders, and families of bacteria were shown in Figure S1.

### 3.3 Alpha diversity

**FIGURE 4:**
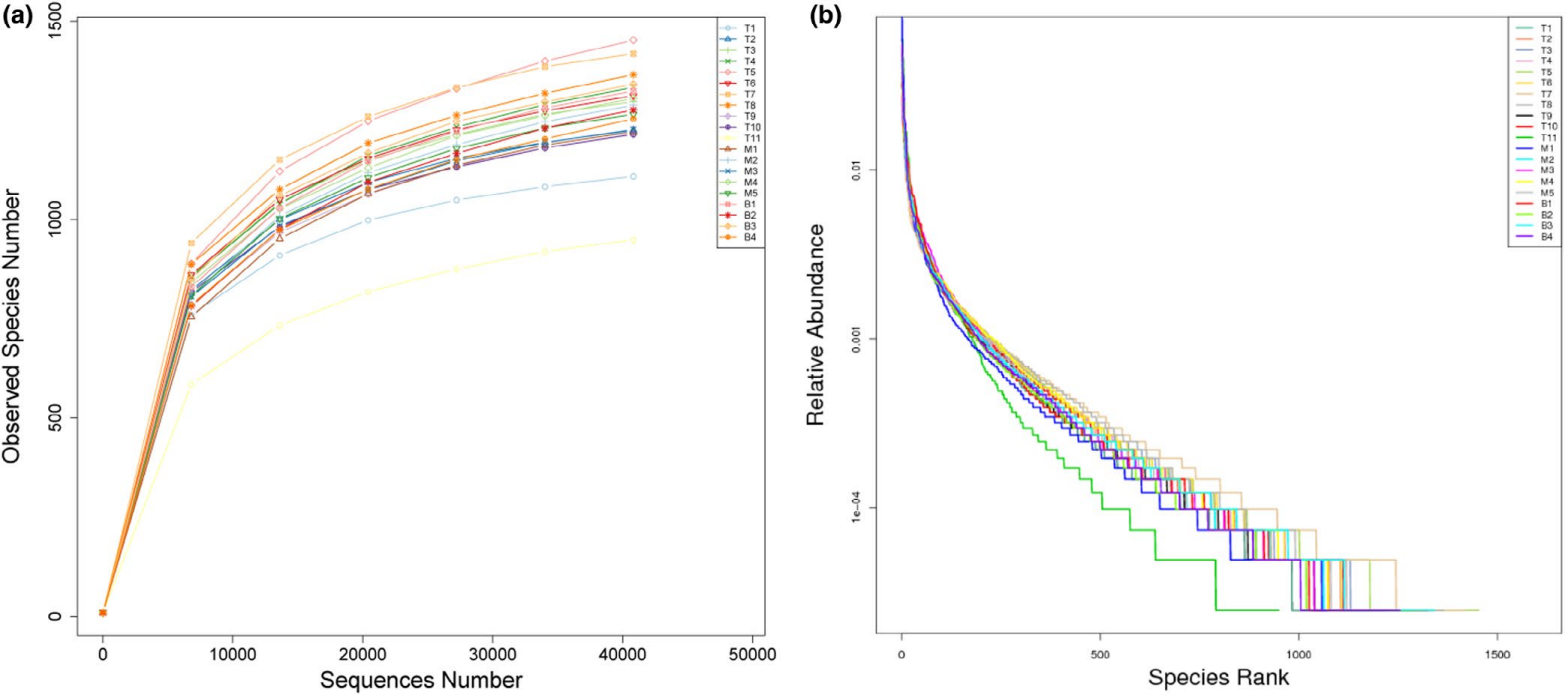
Rarefaction curves (a) and rank‐abundance curves (b) of alpha diversity. The rarefaction curves of observed species tend to approach the saturation plateau. Rank‐abundance curves showing species abundance distributions in three species

Rarefaction curves showed that the sequencing depth was sufficient to cover biodiversity of the samples when approaching the plateau phase (Figure 4a). The rank‐abundance curve reflected species richness and species evenness for intuitive visualization (Figure 4b).

**FIGURE 5:**
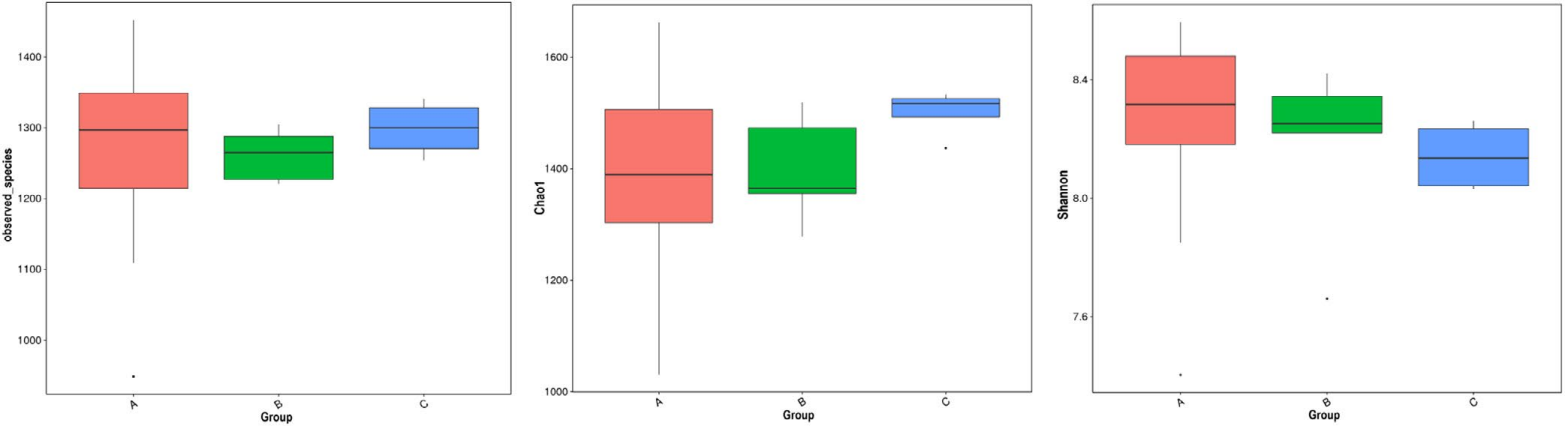
Boxplots of alpha diversity as measured by Observed species, Chao1 richness and Shannon diversity index. No significant differences were observed among the 3 groups for alpha diversity indexes

Alpha diversity metrics (Table 1), such as observed species, Chao1, and Shannon index, were used to characterize the bacterial diversity between groups (Figure 5). However, no significant differences were demonstrated among groups.

**TABLE 1:** Alpha diversity metrics were calculated in fecal samples of gut microbiota from tahr, mouflon, and bharal

Species	Shannon	Simpson	Chao1	ACE	Goods_coverage
*Hemitragus jemlahicus*	8.24	0.99	1,391.32	1,389.95	0.99
*Ovis orientalis*	8.18	0.99	1,398.44	1,397.64	0.99
*Pseudois nayaur*	8.14	0.99	1,501.09	1,472.89	0.99

### 3.4 Correlation in gut microbiota between three species

**FIGURE 6:**
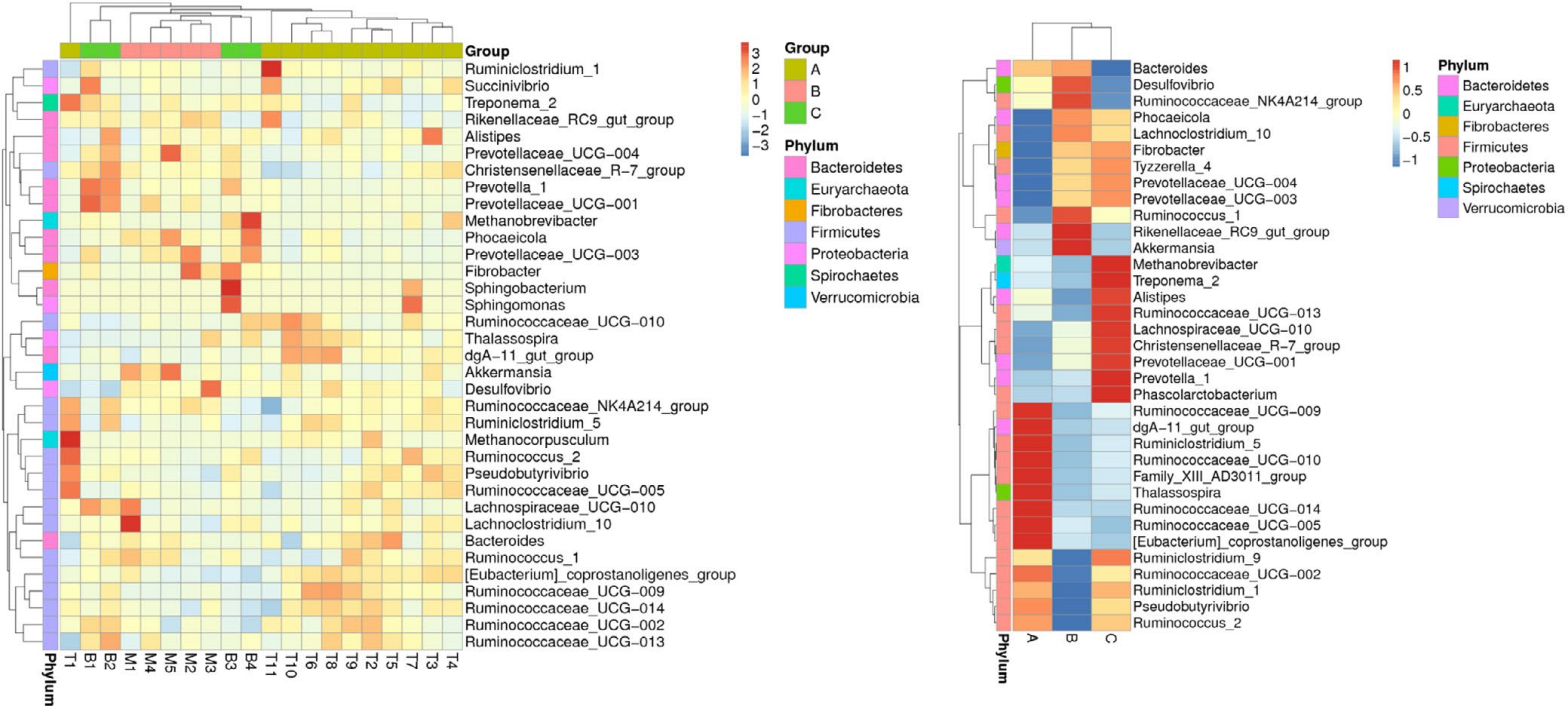
Heat maps of top 35 genera of relative abundance detected among 20 samples in three species. Samples of mouflon cluster closely with bharal

A heat map, based on the relative abundances of the top 35 genera, was generated to visualize the bacterial community composition better at the genus level (Figure 6). The heat map accounted for the microbiota composition and distribution across samples, and samples with more similar bacterial composition and structure clustered closer in phylogenetic relationships. From these results, we can find that most of the members of top 35 genera were classified to phyla Firmicutes and Bacteroidetes. Besides, mouflon was clustered with bharal and then grouped together with tahr, which suggested that structure of gut microbiota was more similar between mouflon and bharal.

### 3.5 Beta diversity of gut microbiota

**FIGURE 7:**
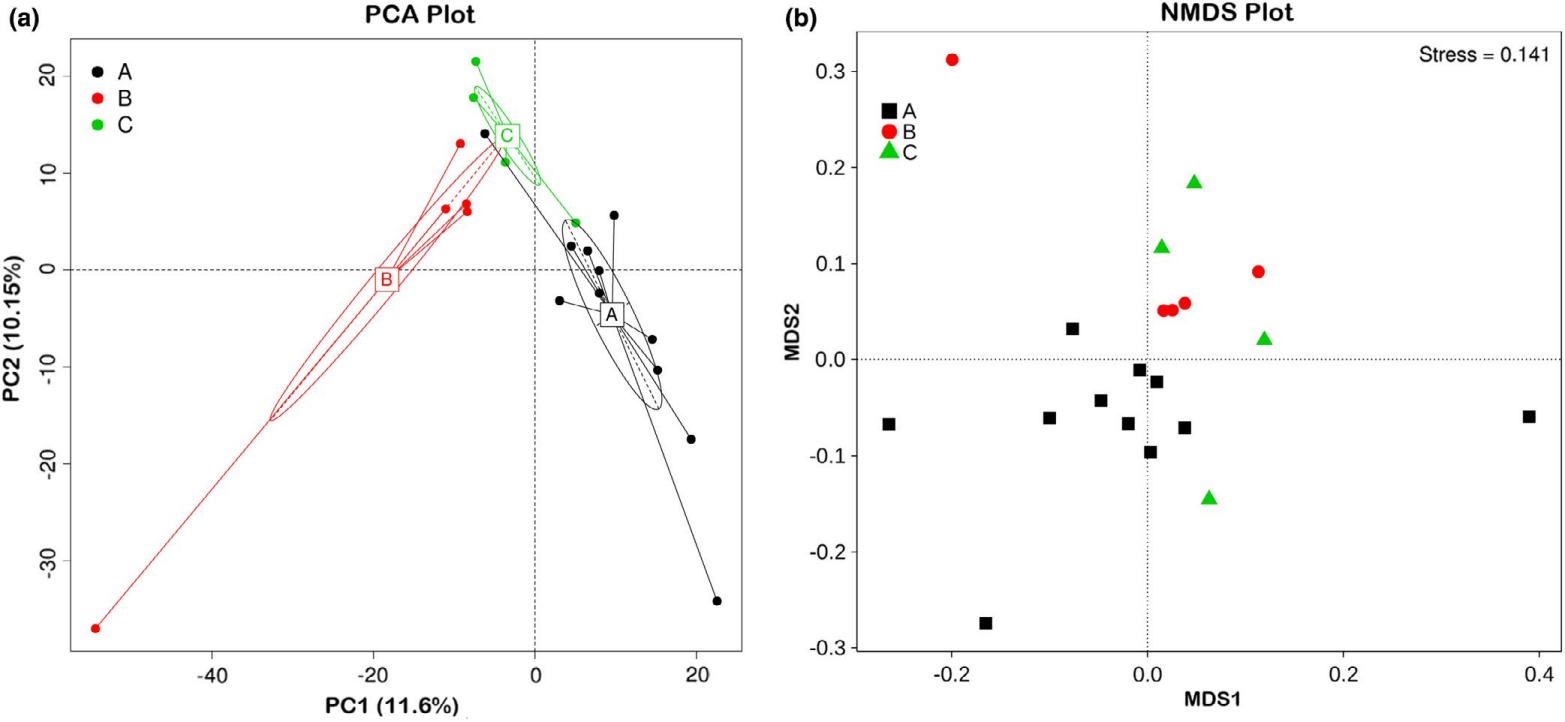
PCA plot (a) and NMDS plot (b) illustrate the separation of samples in three species. PCA reveals samples from different species form distinct clusters, respectively, whereas NMDS plot shows the microbiota from all the groups tend to cluster together

**FIGURE 8:**
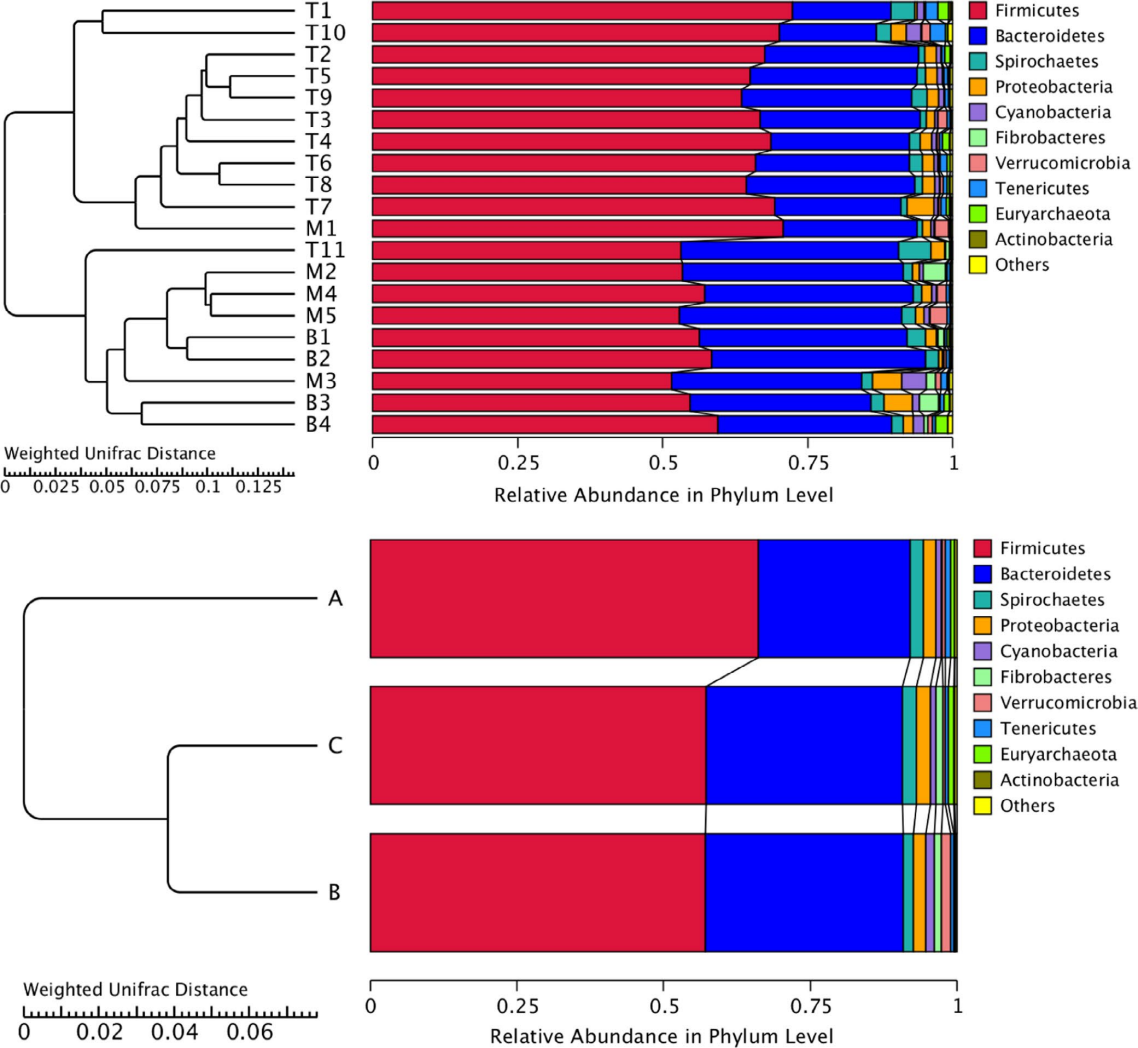
UPGMA dendrograms of samples from three species. UPGMA clustering analysis revealed the gut microbiota composition between mouflon and bharal tends to be more similar compared with tahr at the phylum level

Principal component analysis showed clusters of different groups, differences among three groups were observed (Figure 7a). A difference in bacterial community was also observed by NMDS plot, and the differences appeared to be rather small between mouflon and bharal (Figure 7b). Weighted and Unweighted UniFrac PCoA analysis was carried out to identify discrepancies between groups (Figure S2). Furthermore, UPGMA method was used to quantitatively visualize the similarities in gut microbiota among different species (Figure 8). The UPGMA dendrograms showed that the gut microbial communities of mouflon were more similar to that observed in bharal, with the relative abundance of various phyla.

### 3.6 Statistically difference among groups and biomarkers with significant difference

Statistical significance in the bacterial community structure between groups was analyzed by MRPP and ANOSIM. The difference between tahr and the other two species was significant, while the difference between mouflon and bharal was not significant in the MRPP (Table 2). In addition, the difference between mouflon and bharal was still smaller, although not significant in the ANOSIM (Table 2). This also indicated that the bacterial communities in tahr was more unique, while the gut microbiota in mouflon was more similar to that observed in bharal.

**TABLE 2:** ANOSIM (Analysis of Similarities) and MRPP (Multi‐Response Permutation Procedure) analysis presented overall, and pairwise tests were performed to analyze the significant differences in microbial communities between groups

Groups	ANOSIM		MRPP			
	*R*	*p*	*A*	Observed delta	Expected delta	*p*
A–B	0.287	0.053	0.059	0.349	0.371	0.001
A–C	0.317	0.051	0.030	0.367	0.378	0.011
B–C	0.169	0.070	0.020	0.366	0.374	0.064

Note

*A* > 0 indicated that the difference between groups is greater than that within groups. A: tahr; B: mouflon; C: bharal.

**FIGURE 9:**
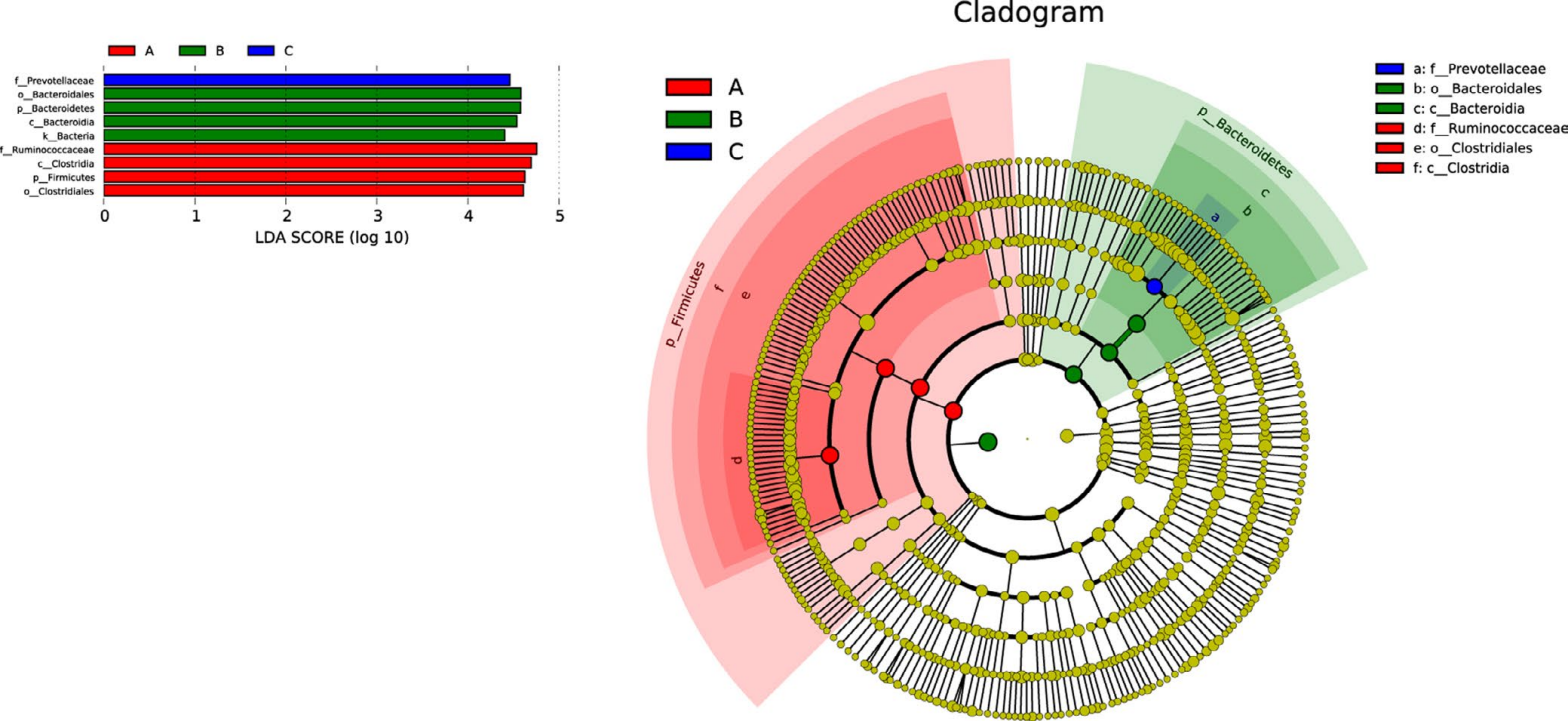
Comparisons of bacteria in three species using LefSe. Biomarkers of different species were identified (LDA score > 4.0, *p* < 0.05), and the cladogram representing the taxonomic hierarchical structure of the identified biomarkers was generated

*LDA* Effect Size analysis (LEfSe, LDA score > 4.0) was used to characterize bacterial features which were specific among three groups (Figure 9). A variety of biomarkers were determined in a total of 20 samples. The phylum *Firmicutes* as a biomarker exhibited a significantly higher relative abundance in tahr (*p* = 0.039). Furthermore, biomarkers from phylum Bacteroidetes to order Bacteroidales were observed in mouflon. Pairwise comparison was also performed between these three species (Figure S3).

## 4 DISCUSSION

Colonization of the gut with a microbiota in hosts may be related to shape and regulate immune system, and an imbalance in intestinal microflora was proved to be associated with various diseases (Ahmadmehrabi & Tang, 2017; Barlow et al., 2015; Salminen et al., 1998). In this study, the intestinal microbial community structures of these three species were examined under the condition of ruling out the potential environmental and diet impact. Accumulating evidence reveals that the gut microbiota is involved in the species phylogenies (Davenport et al., 2017; Moeller et al., 2016; Nishida & Ochman, 2018; Sanders et al., 2014). Moreover, our results showed that host species had a certain impact on the intestinal microbiomes, and it was also supported by the previous studies (Amato et al., 2016; Li et al., 2015). However, the host phylogeny is not always consistent with intestinal microbiota divergence (Li et al., 2018).

Alpha diversity showed no significant differences in our results and indicated that the intestinal microflora structure was similar among samples. (Ley et al., 2008; Li et al., 2018). The predominant phyla in three different herbivore species were Firmicutes and Bacteroidetes, accounting for more than 90% of the intestinal flora and the result was consistent with those obtained in other studies (Chi et al., 2019; Kong et al., 2014; Ley et al., 2008; Sun et al., 2016). This is a common feature observed for herbivorous ungulates, since these two phyla play a crucial role in fiber and carbohydrate degradation (Bird et al., 2019; Brulc et al., 2009; Fernando et al., 2010). The relative abundance of the phyla ranked in top 5 from three species accounting for more than 96%. Further, the phyla ranked in top 4 were consistent among the three species, and the proportion was estimated over 94%. This finding seems to be common in herbivore (Mu et al., 2019; Stalder et al., 2019; Miglior, 2019; Zhao et al., 2016). Interestingly, prior study on wild and captive bharal have demonstrated that statistically significant differences of the intestinal microbiota of phyla Firmicutes and Bacteroidetes were found between the captive and the wild bharal (Chi et al., 2019), and our results seemed to be more similar to those lived in wild when compared with the relative abundance of the phyla Firmicutes and Bacteroidetes. Additionally, the results indicated that the Firmicutes/Bacteroidetes (F/B) ratio was notable differences between tahr and other two species, with a higher ratio in tahr. Considering the important roles of the high ratio in intestinal microbiota‐mediated energy absorption and maintaining host energy balance (Lan et al., 2017; Zhang et al., 2016), we can infer that tahr possessed a stronger ability to absorb and store energy from the diet.

At the family level, the relative abundance of dominant microbial species microbes showed broad consistency between the three species. For instance, Ruminococcaceae, Lachnospiraceae, Rikenellaceae, Prevotellaceae, and Bacteroidaceae were dominant families in previous study, which were also represented in our dataset (AlZahal et al., 2016; Bird et al., 2019). Of note, Ruminococcaceae and Lachnospiraceae belonging to the phylum Firmicutes were the highest relative abundance of families among the three species, whereas the remaining families belonged to the phylum Bacteroidetes. The families of Lachnospiraceae and Ruminococcaceae belonging to order Clostridiales are known to have a wide range of metabolic abilities, including proteolytic, saccharolytic, and cellulolytic activity, which could be related to volatile fatty acids (VFAs) production (Biddle et al., 2013; Blasco et al., 2020; Brulc et al., 2009). The bacteria families (Bacteroidaceae, Rikenellaceae, and Prevotellaceae) belonging to the Bacteroidetes phylum, dominated in the three species fecal microbiome had been characterized as the main source of carbohydrate active enzymes (CAZymes) of the rumen microbiome in cattle, and considered to be primary polysaccharide decomposition bacteria in many microecosystems (Flint et al., 2012; Gong et al., 2020; Jose et al., 2017). Furthermore, the high abundance of these families, were consistent with other animals, should be the core and stable microbial community to herbivores, and played roles in food digestion and short‐chain fatty acids (SCFAs) production (Budden et al., 2017; Louis & Flint, 2009).

Studies indicated that bacterial microbiomes can maintain a similarity and relatively stable between wild and captive condition (Alfano et al., 2015; Jesús‐Laboy et al., 2012). With supporting evidence from prior research, the family Ruminococcaceae, proposed as an enterotype identifier and core taxa, showing variation in the relative abundance among individuals (Falony et al., 2016). Interestingly, a recent study suggested that Ruminococcaceae and Lachnospiraceae exhibited lower abundance in captive gaurs (Prabhu et al., 2020), while relative abundance of them were the highest in our results. In addition, the Fibrobacteres bacteria, which is a small bacterial phylum and related to fermenting dietary fiber, was predominantly abundant in bovine rumen (Li et al., 2020), and the relative abundance of Fibrobacteres in tahr was significantly lower than those in mouflon and bharal. A possible explanation for this might be that the mouflon and bharal, with notable lower F/B ratio, may increase the rate of fiber digestion to fill their expected energy requirement.

LEfSe analysis showed that several taxa were determined as candidate biomarkers to discriminate the groups. In the present study, Ruminococcaceae, Clostridia, Firmicutes, and Clostridiales were the biomarkers of tahr due to their high relative abundance. Similarly, Bacteroidetes and Bacteroidia were the biomarkers of mouflon, while family of Prevotellaceae was the featured bacteria in bharal. One interesting finding was that the relative abundance of genus Akkermansia was significantly higher in mouflon when compared with tahr and bharal, and it has been proved that Akkermansia could help in mucin‐digestion, which showed a positive correlation between metabolic ability and host health.

Although all animals were kept under the same conditions, the results revealed that shared OTUs account for 74.42% in tahr, 83.57% in mouflon, and 83.10% in bharal, respectively. In addition, we also found that the tahr showed a larger number of unique OTUs, and the larger amount of shared OTUs were detected between tahr and bharal when conducted pairwise comparisons. Furthermore, the weighted UPGMA phylogenetic trees were constructed based on the complexity and abundance of the commensal flora with the top 10 ranked phyla, and the results showed that the mouflon and bharal samples clustered closer together and were basically consistent with the results from the hierarchical clustering heat map. However, previous studies have shown that a closer phylogenetic relationship between the species of bharal and tahr than mouflon (Soria‐Carrasco & Castresana, 2012; Toljagić et al., 2018). Of note, the intestinal bacteria play a critical role in host metabolism and the moderate correlation has been demonstrated between hosts' phylogenies and the composition of gut communities (Brooks et al., 2016; Loo et al., 2019), but a substantial majority of studies could not exclude effects, generated by external factors, due to the lack of controlling external environment conditions (Laviad‐Shitrit et al., 2019). For example, giant and red pandas, which preferentially feed on bamboo, harbor a lower intestinal microbial diversity than other carnivores (Guo et al., 2020). Moreover, hierarchical clustering for gut microbiota demonstrated that the giant panda differed from the red panda and was clustered together with black bear (Tang et al., 2020). However, a recent study on Cervinae under the same condition suggested that the clustering dendrogram of microbiota was not completely consistent with phylogenies of species (Li et al., 2018). We therefore speculated that a correlation between the species' phylogeny and their gut bacterial community hierarchical dendrogram was surely present, but not always be concordant.

Normally, herbivores possess a higher level of diversity than other mammals. The present study furthered our understanding of gut microbiota in the subfamily Caprinae and assessed the correlative relationship between gut microbial diversity and host species. Our results demonstrate that no significant differences in alpha diversity were observed between the three species. A significantly higher ratio of F/B was detected in tahr, and it is usually associated with microbiota‐mediated energy absorption and maintaining host energy balance. Also, samples from mouflon and bharal clustered closer together based on the intestinal microbiota divergence analysis, which is not consistent with the host phylogeny. The results presented here have enabled us to gain deeper insights into the gut microbiota in herbivores, especially for ruminates, and provide strategies for feeding management and protection from disease challenge.

## CONFLICT OF INTEREST

No potential conflict of interest was reported by the authors.

## AUTHOR CONTRIBUTIONS

**Guolei Sun:** Formal analysis (equal); Investigation (equal); Methodology (equal); Software (equal); Writing‐original draft (equal). **Tian Xia:** Formal analysis (equal); Investigation (equal); Software (equal). **Qinguo Wei:** Data curation (equal); Formal analysis (equal); Writing‐review & editing (equal). **Yuehuan Dong:** Formal analysis (equal); Software (equal). **Chao Zhao:** Data curation (equal); Software (equal). **Xiufeng Yang:** Formal analysis (equal); Visualization (equal). **Lei Zhang:** Data curation (equal). **Xibao Wang:** Formal analysis (equal); Software (equal). **Weilai Sha:** Conceptualization (equal); Data curation (equal). **Honghai Zhang:** Conceptualization (equal); Methodology (equal); Supervision (equal); Writing‐review & editing (equal).

## Supporting information

Figure S1

Figure S2

Figure S3

Supplementary Material

## Data Availability

The data sets generated for this study can be found in the SRA database of NCBI, SRA accession: PRJNA664214, PRJNA511517.
