# Supplementary figures and images for: Analysis of gut microbiota in three species belonging to different genera (Hemitragus, Pseudois, and Ovis) from the subfamily Caprinae in the absence of environmental variance
Source: Ecol Evol. 2021 Jul 31;11(17):12129–40. doi: 10.1002/ece3.7976 (PMC8427585; doi:10.1002/ece3.7976)

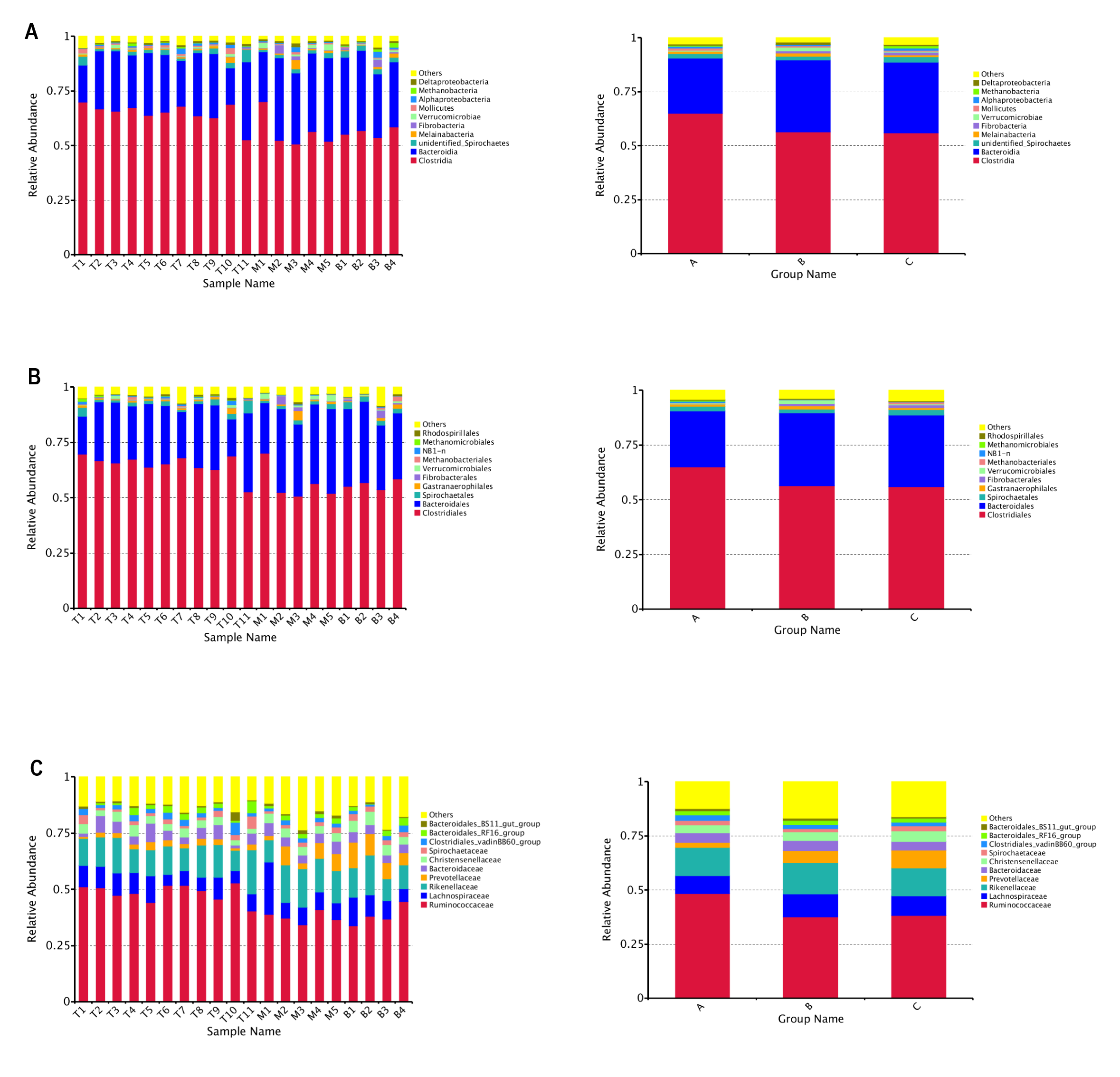

Supplement: Supplementary file 1 — Figure S1 [file ECE3-11-12129-s002.tif]

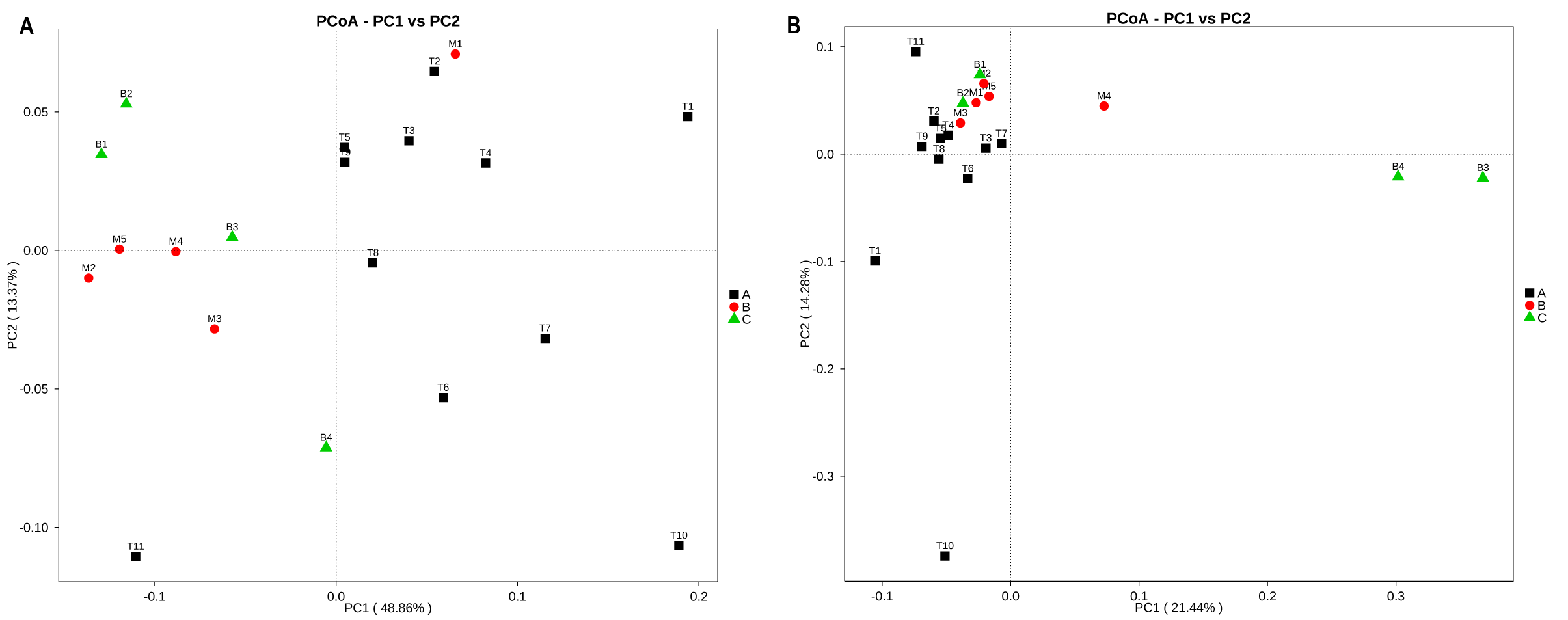

Supplement: Supplementary file 2 — Figure S2 [file ECE3-11-12129-s003.tif]

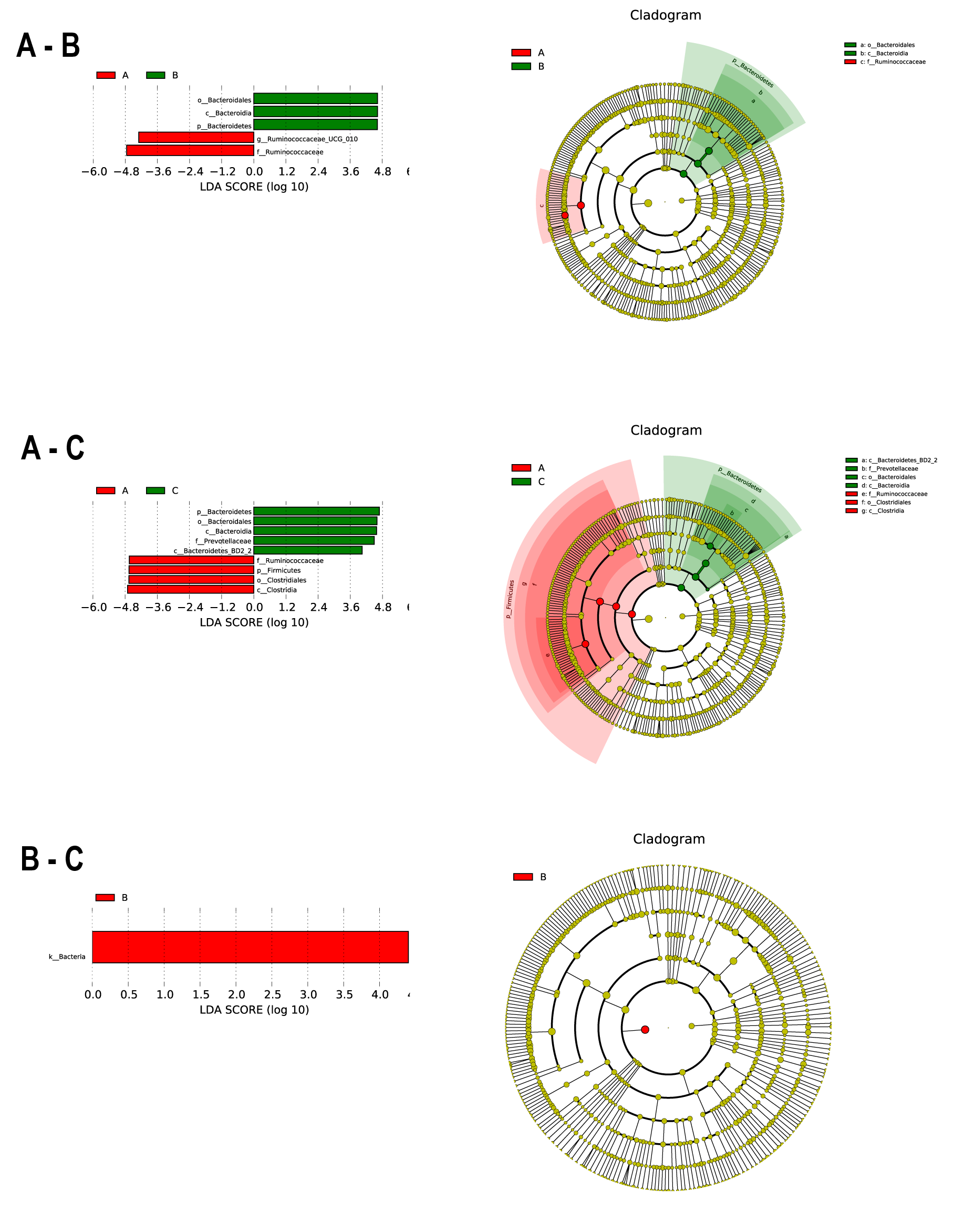

Supplement: Supplementary file 3 — Figure S3 [file ECE3-11-12129-s001.tif]
