# Supplementary material for: Analysis of gut microbiota in three species belonging to different genera (Hemitragus, Pseudois, and Ovis) from the subfamily Caprinae in the absence of environmental variance
Source: Ecol Evol. 2021 Jul 31;11(17):12129–40. doi: 10.1002/ece3.7976 (PMC8427585; doi:10.1002/ece3.7976)
Supplement: Supplementary file 4 — Supplementary Material [file ECE3-11-12129-s004.docx]

**SUPPROTING INFORMATION**

Additional supporting information may be found in the online version of this article:

**Supplemental Figure S1** Relative abundance of the top 10 classes (A), orders (B), and families (C) of bacteria.

**Supplemental Figure S2** PCoA plots of microbial communities with weighted (A) and weighted (B) UniFrac distances among different samples.

**Supplemental Figure S3** Pairwise comparison of bacteria in three species by LEfSe analysis.
